# Efficacy of aquatic exercise in chronic musculoskeletal disorders: a systematic review and meta-analysis of randomized controlled trials

**DOI:** 10.1186/s13018-023-04417-w

**Published:** 2023-12-08

**Authors:** Tianyue Wang, Jiamin Wang, Yuheng Chen, Yanmin Ruan, Senjie Dai

**Affiliations:** 1 The Second Clinical Medical College, Zhejiang Chinese Medical University, Hangzhou, 310053 China; 2 The Fourth Clinical Medical College, Zhejiang Chinese Medical University, Hangzhou, 310053 China

**Keywords:** Water exercise, Musculoskeletal pain, Sports medicine, Rehabilitation medicine

## Abstract

**Background:**

Aquatic exercise (AE) is becoming ever more popular as a physical therapy, while it is unclear what precise improvements it will produce and how effective it will be in comparison with other non-surgical therapies. The study aimed to assess whether AE positively impacts chronic musculoskeletal disorder patients in terms of pain, physical function, and quality of life.

**Methods:**

PRISMA guidelines were followed, and our study protocol was published online at PROSPERO under registration number CRD42023417411. We searched PubMed, Embase, Web of Science, and Cochrane library databases for English-language articles published before April 11, 2023, including studies from all relevant randomized controlled trials (RCTs). After screening, we ultimately included 32 RCTs with a total of 2,200 participants. We also performed subgroup analyses for all included studies. This meta-analysis calculated standardized mean difference (SMD) with 95% confidence interval (CI), and the variance was estimated using a random-effects model. The quality of the included studies was assessed by using the Cochrane collaborative "risk of bias" assessment tool (version 2.0). Thus ensuring that the literature included is of high quality.

**Results:**

This meta-analysis included 32 trials with 2,200 participants; these patients were all between the ages of 38–80. The study showed that compared to the no exercise (NE) group, patients in the AE group experienced a remarkable reduction in pain (SMD: -0.64, *P* < 0.001), a significant increase in physical function (SMD: 0.62, *P* < 0.001), and a statistically significant improvement in quality of life (SMD: −0.64, *P* < 0.001). When compared to land-based exercise (LE), AE significantly relieves patients' pain (SMD: −0.35, *P* = 0.03).

**Conclusions:**

This is the first systematic review and meta-analysis to study whether AE could improve chronic musculoskeletal disorders. The evidence suggests that AE benefits pain, physical function, and quality of life in adults with chronic musculoskeletal conditions compared to NE. Furthermore, when compared to LE, AE continues to provide a better improvement in patient pain. More long-term clinical trials are needed to confirm AE's positive effects and improvement mechanisms and the more existential advantages compared to LE.

**Supplementary Information:**

The online version contains supplementary material available at 10.1186/s13018-023-04417-w.

## Introduction

Chronic musculoskeletal disorders are the second leading cause of disability, with approximately 1.71 billion people worldwide suffering from the disease to date [1]. Per the 11th revision of the International Classification of Diseases and Related Health Problems [2], the definition of chronic musculoskeletal disorder is a persistent disorder lasting more than 3 months and is typically characterized by chronic pain and functional disability [3]. Patients often show stiffness and pain in joints and muscles, injury, and inflammation in related parts of the body [4]. In severe cases, complications, such as hypertension and depression, make patients' survival less efficient and greatly limited, as well as imposing a heavy burden on society and the economy [1]. For this reason, the therapy of chronic musculoskeletal disorders has always been the focus of attention of patients, the clinical and scientific community, and society.

In the traditional conservative treatment of chronic musculoskeletal disorders, drugs, injections, or electroshock therapy are often costly [5] and the results are not particularly gratifying to patients [6]. Fortunately, aquatic exercise (AE) may bring new treatment options for patients. AE refers to water-based therapy or training [7]. In the last 20 years, AE has become increasingly popular as an emerging physical therapy for patients and physicians alike [8]. It has the unique advantage of being less costly while meeting the corresponding psychosocial needs and reducing the patient's feelings of helplessness and isolation. At the same time, since the patients involved are mainly middle-aged and elderly [9], a special group with a higher risk of falling, and the buoyancy generated by water can reduce the possibility of injury to the participants. Moreover, as buoyancy reduces the pressure of gravity on muscles and joints, it is also more suitable for special groups such as obese, postmenopausal women, and injured athletes [10].

In recent years, a large number of clinical trials have been conducted on the topic of AE, relevant research found that AE has the potential to improve the treatment of chronic musculoskeletal diseases, such as the improvement of pain and quality of life. However, to date, while some studies have described AE as an effective treatment for osteoarthritis and fibromyalgia by improving pain and quality of life, there are still studies concluding that AE is not effective [11–13]. Meanwhile, we found that these studies were limited to a specific disease [14]. Of the relevant meta-analyses currently available, the systematic review by McVeigh et al*.* [15] and Waller et al*.* [16] had the drawback of including a small number of articles due to the early years of the study. The study by Heywood et al*.* [17] could not evaluate the therapeutic effects of AE in a comprehensive and multifaceted manner due to the variable quality of the included articles and the limitations of the observed indicators, the research analysis by Batterham et al*.* [18] and Lu et al*.* [19] focused on only one type of disease within musculoskeletal disorders and did not summarize this general group of disorders. Besides that, Zão et al*.* [20] only performed a systematic review and did not perform a meta-analysis. There have been no studies using meta-analysis methods to assess whether AE could improve chronic musculoskeletal disorders. The purpose of this study is to investigate the efficacy and role of AE in the treatment of chronic musculoskeletal disorders. We also hope to provide a reference for future clinical applications.

## Methods

### Protocol and registration

The present systematic review and meta-analysis have been completed for registration in PROSPERO (No. CRD42023417411) and followed up the standard Preferred Reporting Items for Systematic Reviews and Meta-Analyses (PRISMA) guidelines and the Cochrane Handbook to ensure a transparent review. The prior research question and search strategy were formulated according to the Population, Intervention, Control/Comparison, and Outcome (PICO) framework to enhance search precision and ensure extensive data extraction to be representative and unbiased. The research question was: Whether AE could improve chronic musculoskeletal disorders?

### Search strategy

The authors systematically searched four databases, PubMed, Embase, Web of Science, and Cochrane Library, for articles published in English before April 11, 2023, and screened all retrieved articles based on the inclusion criteria. We mainly used the following combinations of Mesh terms for the literature search: (hydrotherapy OR aquatic therapy) AND musculoskeletal diseases AND chronic disease. In the meantime, we also screened references in relevant reviews and meta-analyses to avoid omitting qualified articles. The detailed search strategy and results can be shown in Additional file 1: Table S1.

### Inclusion and exclusion criteria

Inclusion criteria were as follows: (1) article type is randomized controlled trial (RCT); (2) participants had to be diagnosed with at least one musculoskeletal disorder that persisted for more than three months, with no age restriction; (3) the experimental group used AE therapy (AE interventions included any such as endurance, resistance, strength, balance, flexibility training in water, and warm-up aerobic exercise), while the control group was studied with land-based exercise (LE) therapy or no exercise (NE) (including non-activity, such as education, and meditation); and (4) the study had to measure the baseline values of each indicator for each group of subjects before the intervention and the corresponding values at the end of the intervention, with some data from studies with follow-up at the end of the intervention, were also included, measuring indicators including pain, physical function and quality of life.

The exclusion criteria were as follows: (1) articles published in languages other than English; (2) studies without a control group; (3) articles that only mentioned that the participants were in the state of being in the preoperative phase of joint replacement or had completed the relevant surgery, but did not mention the specific diagnosed disease of the participants; (4) the experimental group used other therapies, such as spa therapy and balneotherapy; (5) the experimental group engaged in AE along with LE; (6) the control group used any therapy other than LE and no sports that might have had some effect on the physical fitness of the participants; (7) full text or complete data are not available from relevant sources and the relevant data mentioned in the article is not available; and (8) duplicate published studies.

### Data extraction

The two authors (JW and YC) independently followed a pre-designed table for the extraction of relevant data from the screened articles. Any problems that arose during the extraction of the data have been resolved after a thorough discussion between the authors. Data extracted by the authors from each study included article authorship, date of publication, participants' diagnosis, demographic characteristics (number of participants, gender, age), intervention characteristics (intervention-specific measures, intervention period, and frequency), baseline and post-intervention outcome data (major endings include pain, physical function, and quality of life), the types of data included were mean, standard deviation (SD), and sample size (if the study had no SD, quartiles or 95% confidence interval (CI) or standard error (SE) or standard error of the mean (SEM) were included and converted to SD by a conversion formula). In the case of multiple assessment scales for the same indicator in the included outcome data, preference was given to the scale used for the primary outcome or to the more well-known and universal scale. Table 1 represents the list of outcome measures that met the inclusion criteria.

**Table 1:** Outcome measures eligible to be included in the meta-analysis

Outcomes	Scales
Pain	VAS, SF-36, FIQ, WOMAC, KOOS, BPI, HAQ, MPQ
Physical function	SF-36, SF-12, FIQ, WOMAC
Quality of life	FIQ, KOOS, HAD, BAI, ODI, PQOL, SF-36, HAQ, AQOL, EQ-5D, WOMAC

^VAS, Visual Analog Scale; SF−36, Medical Outcomes Study 36−Item Short−Form Health Survey; FIQ, Fibromyalgia Impact Questionnaire; WOMAC, Western Ontario and McMaster Universities Osteoarthritis Index; KOOS, Knee Injury and Osteoarthritis Outcome Score; BPI, Brief Pain Inventory; HAQ, Health Assessment Questionnaire; MPQ, McGill Pain Questionnaire; SF−12, Medical Outcomes Study 12−Item Short−Form Health Survey; HAD, Hospital Anxiety and Depression Scale; BAI, Beck Anxiety Inventory; ODI, Oswestry Disability Index; PQOL, Perceived Quality of Life Scale; AQoL, Arthritis Quality of Life Scale; EQ−5D, European Quality of Life−5 Dimensions Scale.^

### Quality assessment and risk of bias

Two reviewers (JW and YC) used the Cochrane collaborative "risk of bias" assessment tool to conduct a bias analysis of the included randomized controlled studies. It contains a total of six domains: selective bias (random sequence generation, allocation concealment), implementation bias (subject, trial personnel unblinding), measurement bias (outcome assessor unblinding), follow-up bias (incomplete outcome data), reporting bias (selective reporting of results), and other bias (other factors causing risk of bias). The risk of bias was ranked into three levels: low risk, high risk, and unknown risk. The criteria for determining each risk were those documented in the Cochrane Handbook for the Systematic Evaluation of Interventions. In the event of disagreement between two reviewers when evaluating the same study, a third reviewer (TW) was invited to conduct the evaluation.

### Statistical analysis

The authors used Review Manager software (version 5.3) to analyze the effects of AE on patients with chronic musculoskeletal disorders, measuring pain, physical function, and quality of life. Pain, physical function, and quality of life were analyzed as subgroups according to the type of diseases the patients had (osteoarthritis, fibromyalgia, low back pain, and ankylosing spondylitis). Each study established at least one control group, and the effect of the intervention was assessed by comparing baseline and post-intervention values between the test and control groups. Data from each group were combined and meta-analyzed, described using standard mean difference (SMD) and 95% CI, and the authors used chi-square tests to examine heterogeneity and *I*^2^ to assess the effect of heterogeneity. Substantial heterogeneity was considered to exist if *I*^*2*^ ≥ 50%. The effects were considered negligible for SMD < 0.2, small for 0.2 ≤ SMD < 0.5, moderate for 0.5 ≤ SMD < 0.8, and high for SMD ≥ 0.8. Considering the existence of different studies using different scales when measuring the same indicator, a random-effects model was selected and *P* < 0.05 was considered statistically significant. Potential factors that lead to possible heterogeneity, i.e., disease type, were investigated by subgroup analysis. We also used the software to create funnel plots for the stability of studies and performed sensitivity analyses to identify possible sources of heterogeneity by excluding included studies on a study-by-study basis to observe changes in heterogeneity.

## Results

### Study selection

**Fig. 1:**
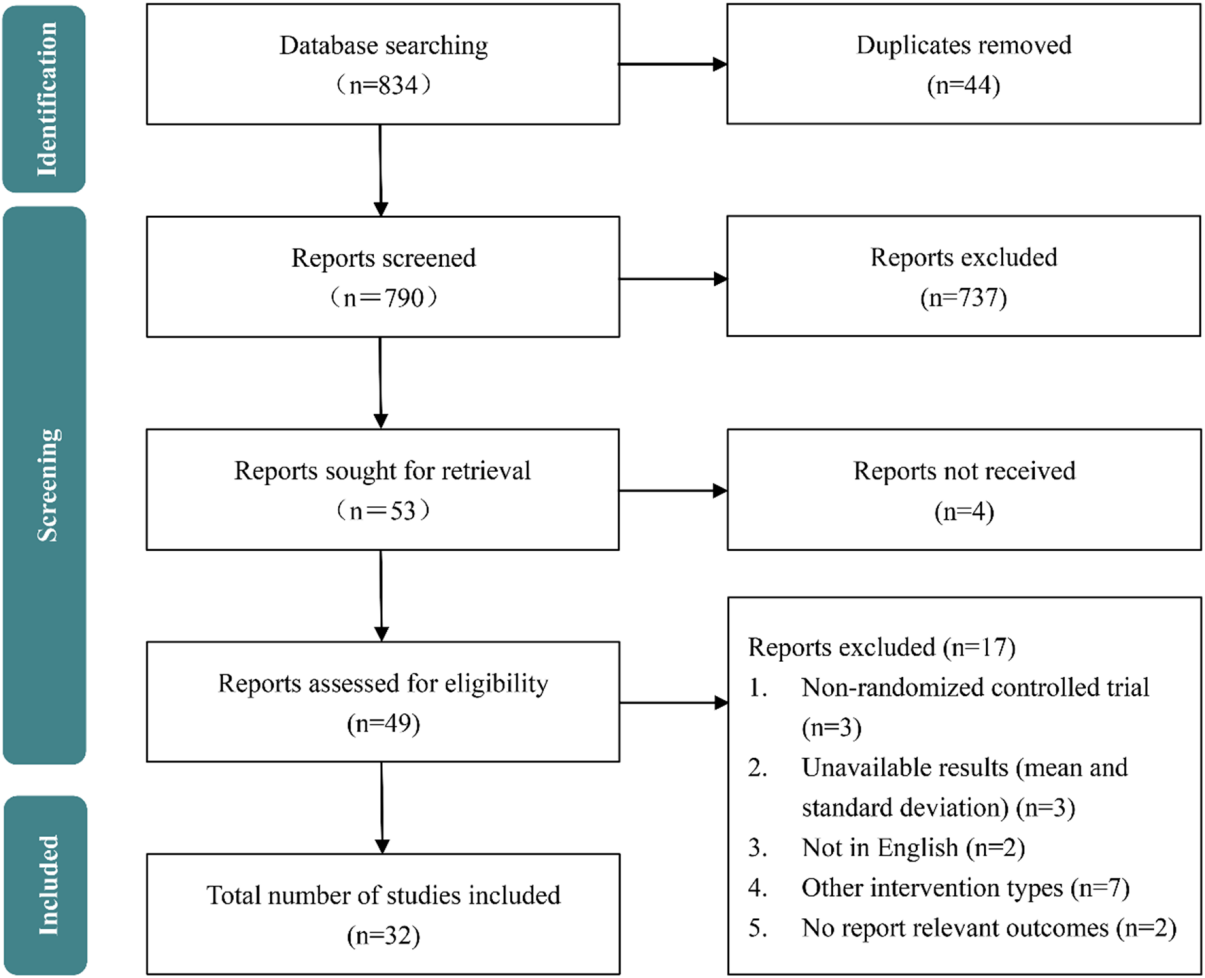
Flowchart of the selection of studies included in the meta-analysis

The research provided a detailed PRISMA flow chart in Fig. 1. According to our search strategy, we searched 834 articles from four databases. After removing duplicate articles and some articles that were not relevant to the subject, the remaining 53 articles were for full-text review. Then, 4 reports were not accessible and 17 studies were excluded for various reasons. Finally, 32 studies were included in this meta-analysis.

### Study characteristics and quality assessment

This meta-analysis included 32 RCTs [9, 11–13, 21–48], relating 2,200 subjects with an approximate age of 38–80. Table 2 shows the training periods of AE ranged from 3 to 32 weeks and the training frequencies from 1 to 5 sessions per week. In terms of training time for AE, most experiments set it from 30 to 60 min. Most studies reported outcome measures of pain (93.75%), physical function (62.5%), and quality of life (68.75%). The quality assessment of RCTs is shown in Additional file 1: Fig. S1. Overall, in randomized studies, the low risk was dominant in the three key indicators. However, as some articles lacked key information on the risk of bias, these articles were flawed by an unknown bias. Additionally, these unspecified risks of bias exist mainly due to participant and personnel blinding, allocation concealment, and the blinding of outcome.

**Table 2:** Characteristics of the included trial

Author	Year of publication	Disease	Subject (age: Mean + SD)	Intervention methods	Intervention Period	Intervention time	Outcomes
Alkatan et al	2016	Osteoarthritis	40 subjects	AE + LE	12 weeks [3/week]	AE:20–45'Intervention LE:20–45'Intervention	Pain; physical function; quality of life
			AE(N = 20)				
			LE(N = 20)				
Andrade et al	2018	Fibromyalgia	54 F AE(N = 27) 48 ± 8 NE(N = 27) 47 ± 8	AE + NE	16 weeks [2/week]	AE: 45'Intervention	Pain; physical function; quality of life
Assis et al	2006	Fibromyalgia	60 F AE(N = 30) 43.43 ± 10.76 LE(N = 30) 42.17 ± 10.05	AE + LE	15 weeks [3/week]	AE:10'W + 40 MW + 10'Str LE:10'W + 40 MW + 10'Str	physical function; quality of life
Baena-Beato et al	2014	Low back pain	38 subjects AE(N = 21) 50.9 ± 9.6 12F + 9 M NE(N = 17) 46.2 ± 9.8 10F + 7 M	AE + NE	2 months [5/week]	AE:10'W + 35–45'MW + 10'str	Pain; physical function; quality of life
Belza et al	2002	Osteoarthritis	157 subjects AE(N = 36) NE(N = 121)	AE + NE	20 weeks [2/week]	AE: 1 h Intervention	Pain; quality of life
Britto et al	2020	Fibromyalgia	33 F AE(N = 16) 50.25 ± 6.09 LE(N = 17) 46.18 ± 10.84	AE + LE	8 weeks [3/week]	AE:10'W + 40'MW + 10'Str LE:10'w + 40'MW + 10'Str	Pain; physical function; quality of life
Cuesta-Vargas et al	2012	Low back pain	49 subjects AE(N = 25) NE(N = 24)	AE + NE	16 weeks [3/week]	AE:30'Intervention	Pain; physical function
Dundar et al	2009	Low back pain	65 subjects AE(N = 32) 35.3 ± 7.8 LE(N = 33) 34.8 ± 8.3	AE + LE	4 weeks [5/week]	AE:15'W + 40 MW + 5'Str LE:60'Intervention	Pain; physical function
Dundar et al	2014	Ankylosing spondylitis	69 subjects AE(N = 35) 42.3 ± 11.3 5F + 30 M LE(N = 34) 43.1 ± 11.7 6F + 28 M	AE + LE	4 weeks [5/week]	AE:15'W + 40 MW + 5'Str LE:60'Intervention	Pain; physical function
Evcik et al	2008	Fibromyalgia	61 subjects AE(N = 31) 43.8 ± 7.7 LE(N = 30) 42.8 ± 7.6	AE + LE	5 weeks [3/week]	AE:20'W + 35 MW + 5'Str LE:60'Intervention	Pain
Fonseca et al	2019	Fibromyalgia	46 F AE(N = 27) 53.78 ± 10.40 LE(N = 19) 54.47 ± 11.18	AE + LE	10 weeks[1/week]	AE:5'W + 45'MW + 10'Str LE:45'MW	Pain; quality of life
Fransen et al	2007	Osteoarthritis	96 subjects AE(N = 55) 70.0 ± 6.3 40F + 15 M NE(N = 41) 69.6 ± 6.1 34F + 7 M	AE + NE	12 weeks [2/week]	AE:60'Intervention	Pain; physical function
Guillemin et al	1994	Low back pain	102 subjects AE(N = 50) NE(N = 52)	AE + NE	3 weeks	AE:15'Intervention	Pain; quality of life
Gusi et al	2006	Fibromyalgia	34 F AE(N = 17) 51 ± 10 NE(N = 17) 51 ± 9	AE + NE	12 weeks [3/week]	AE:10'W + 40 MW + 10'Str	Pain; quality of life
Hale et al	2011	Osteoarthritis	35 subjects AE(N = 20) NE(N = 15)	AE + NE	12 weeks [2/week]	AE:20'-60'Intervention	Pain; physical function; quality of life
Hinman et al	2007	Osteoarthritis	71 subjects AE(N = 36) 63.3 ± 9.5 24F + 12 M NE(N = 35) 61.5 ± 7.8 24F + 11 M	AE + NE	6 weeks [2/week]	AE:45'-60'Intervention	Pain; physical function; quality of life
Lim et al	2010	Osteoarthritis	75 subjects AE(N = 26) 65.7 ± 8.9 23F + 3 M LE(N = 25) 67.7 ± 7.7 21F + 4 M NE(N = 24) 63.6 ± 5.3 21F + 3 M	AE + LE + NE	8 weeks [3/week]	AE:5'W + 30 MW + 5'Str LE:5'W + 30 MW + 5'Str	Pain; physical function
Lund et al	2008	Osteoarthritis	79 subjects AE(N = 27) 65 ± 12.6 22F + 5 M LE(N = 25) 68 ± 9.5 20F + 5 M NE(N = 27) 70 ± 9.9 20F + 7 M	AE + LE + NE	8 weeks [2/week]	AE:10'W + 30'MW + 10'Str LE:10'W + 30'MW + 10'Str	Pain; quality of life
Mannerkorpi et al	2009	Fibromyalgia	166 F AE(N = 81) 44.6 ± 9.26 NE(N = 85) 46.5 ± 8.30	AE + NE	20 weeks [1/week]	AE:45'Intervention	Pain; physical function; quality of life
McIlroy et al	2017	Osteoarthritis	13 F AE(N = 7) 64.3 ± 8.7 NE(N = 6) 62.3 ± 6.6	AE + NE	6 weeks	AE:30'Intervention	Pain; physical function
Munguía-Izquierdo et al	2008	Fibromyalgia	58 subjects AE(N = 34) 50 ± 7 NE(N = 24) 46 ± 8	AE + NE	16 weeks [3/week]	AE:10'W + 40 MW + 10'Str	Pain; physical function; quality of life
Munukka et al	2016	Osteoarthritis	84 F AE(N = 42) 64 ± 2 NE(N = 42) 64 ± 2	AE + NE	16 weeks [3/week]	AE:60'Intervention	Pain; quality of life
Patrick et al	2001	Osteoarthritis	249 subjects AE(N = 125) 65.7 109F + 16 M NE(N = 124) 66.1 106F + 18 M	AE + NE	20 weeks [2/week]	AE:45–60'Intervention	Pain; quality of life
Sahin et al	2018	Osteoarthritis	59 F AE(N = 30) 60.46 ± 6.82	NE(N = 29) 58.23 ± 7.55	AE + NE 3 weeks [5/week]	AE:45'-60'W	Quality of life
Silva et al	2008	Osteoarthritis	64 subjects AE(N = 32) 59 ± 7.60 30F + 2 M	LE(N = 32) 59 ± 6.08 29F + 3 M	AE + LE 18 weeks [3/week]	AE:50'Intervention LE:50'Intervention	Pain
Sjogren et al	1997	Low back pain	56 subjects AE(N = 28) LE(N = 28)	AE + LE	6 weeks [2/week]	AE:50'Intervention LE:50'Intervention	Pain; physical function
Taglietti et al	2018	Osteoarthritis	60 subjects AE(N = 31) 67.3 ± 5.9 8 M + 23F NE(N = 29) 68.7 ± 6.7 11 M + 18F	AE + NE	8 weeks [2/week]	AE:60'Intervention	Pain; physical function; quality of life
Tomas-Carus et al	2007	Fibromyalgia	34 F AE(N = 17) 51 ± 10 NE(N = 17) 51 ± 9	AE + NE	12 weeks [3/week]	AE: 10'W + 40'MW + 10'Str	Pain; physical function; quality of life
Tomas-Carus et al	2008	Fibromyalgia	30 F AE(N = 15) 50.7 ± 10.6 NE(N = 15) 50.9 ± 6.7	AE + NE	8 months [3/week]	AE:10'w + 50'Intervention	Pain; physical function; quality of life
Tomas-Carus et al	2009	Fibromyalgia	30 F AE(N = 15) 50.7 ± 10.6 NE(N = 15) 50.9 ± 6.7	AE + NE	32 weeks [3/week]	AE:10'W + 50'MW	Pain; physical function
Waller et al	2017	osteoarthritis	87 F AE(N = 43) 63.8 ± 2.4 NE(N = 44) 63.9 ± 2.4	AE + NE	16 weeks [3/week]	AE: 60'Intervention	Pain; quality of life
Wang et al	2010	Osteoarthritis	78 subjects AE(N = 26) 66.7 ± 5.6 22F + 4 M LE(N = 26) 68.3 ± 6.4 23F + 3 M NE(N = 26) 67.9 ± 5.9 22F + 4 M	AE + LE + NE	12 weeks [3/week]	AE:5'W + 40 MW + 5'Str LE:5'W + 40 MW + 5'Str	Pain; quality of life

### Pain

Regarding pain, thirty studies were included in the meta-analysis. Among them, twenty-two studies [9, 11–13, 21–23, 27, 29–33, 35–37, 39–41, 44–46] reported the comparison of AE and NE, eleven studies [11, 13, 25, 26, 28, 30, 34, 38, 42, 47, 48] reported the comparison of AE and LE, and only three studies [11, 13, 30] compared three interventions.

**Fig. 2:**
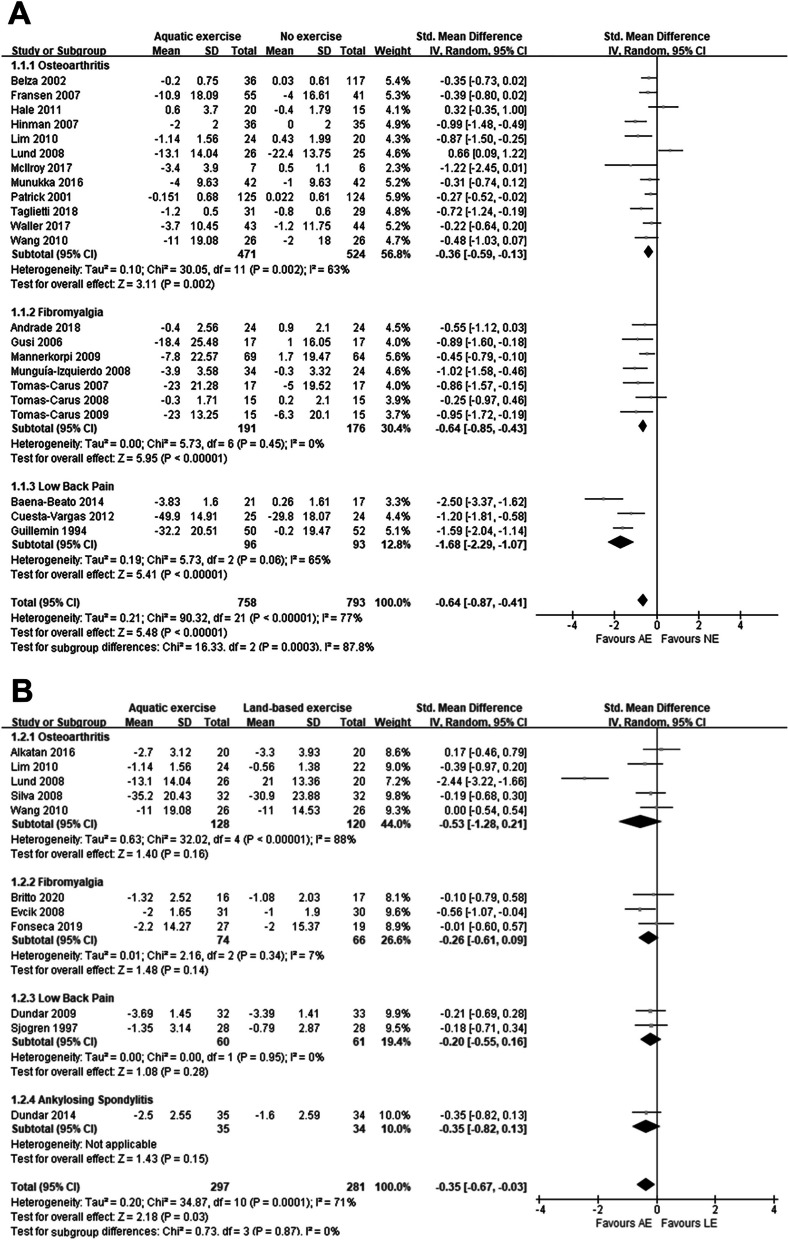
Forest plot of pain outcomes **A** AE versus NE; **B** AE versus LE

From Fig. 2A, we found that AE was effective in reducing participants' pain as compared to NE (SMD: -0.64, *P* < 0.001). Further subgroup analysis of the various musculoskeletal disorders classifications showed that AE had a significant therapeutic effect on osteoarthritis (SMD: −0.36, *P* = 0.002), fibromyalgia (SMD: -0.64, *P* < 0.001) and low back pain (SMD: −1.68, *P* < 0.001).

Also, compared to LE, we found that AE could significantly relieve patients' pain (SMD: −0.35, *P* = 0.03) (Fig. 2B).

### Physical function

Regarding physical function, twenty studies were included in the meta-analysis. Among them, fourteen studies [12, 21–23, 29–31, 33, 37, 39–41, 44, 45] reported the comparison of AE and NE, seven studies [28, 30, 34, 42, 43, 47, 48] reported the comparison of AE and LE, and only one study [30] compared three interventions.

**Fig. 3:**
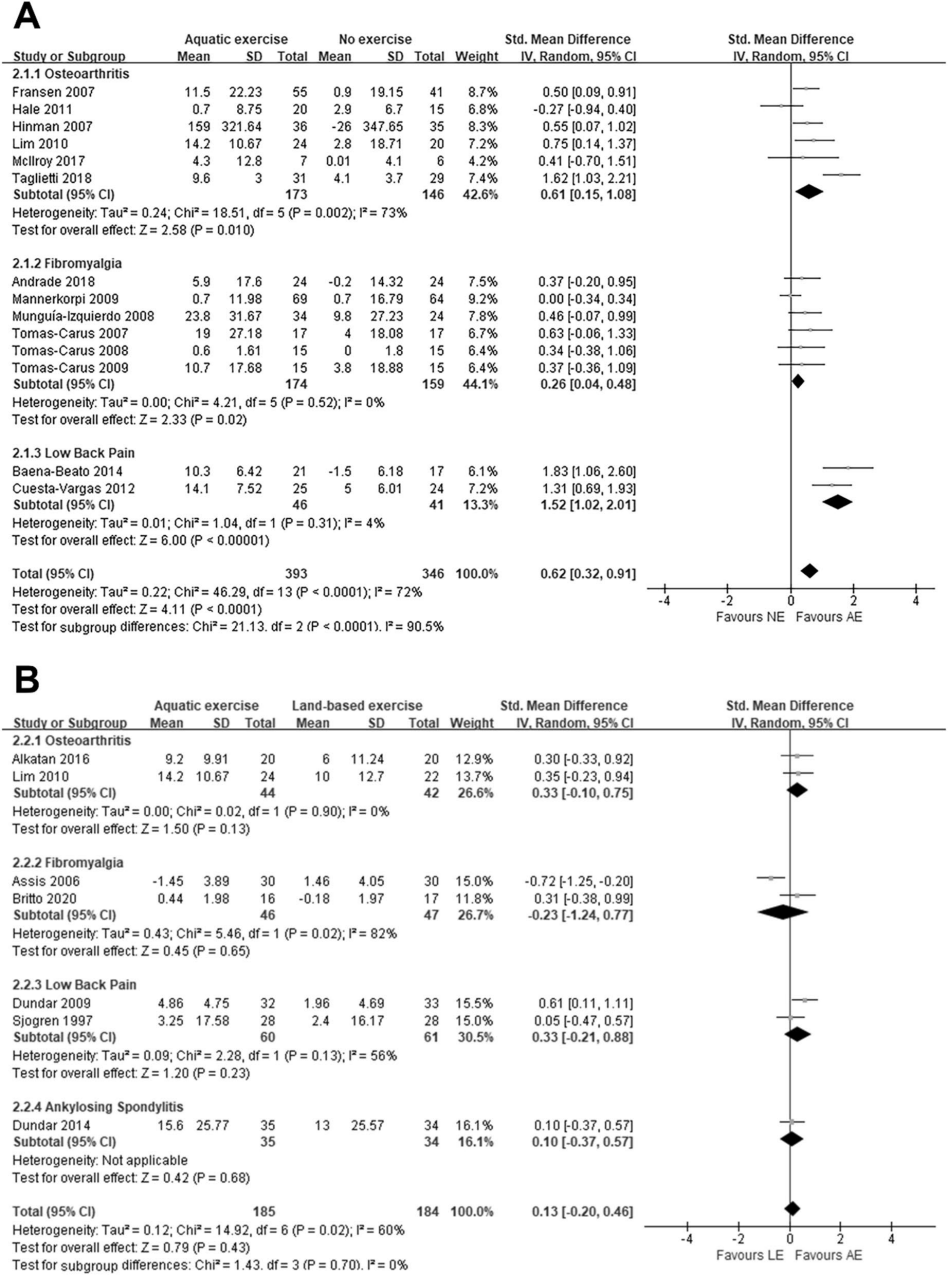
Forest plot of physical function outcomes **A** AE versus NE; **B** AE versus LE

From Fig. 3A, we can see that the patients involved in AE had a significant improvement in physical function compared to NE (SMD: 0.62, *P* < 0.001). Among the various subgroups of AE compared with NE, we found significant treatment effects for osteoarthritis (SMD: 0.61, *P* = 0.01), fibromyalgia (SMD: 0.26, *P* = 0.02), and low back pain (SMD: 1.52, *P* < 0.001).

Interestingly, after analyzing the treatment capacity of AE and LE, we found that for the various musculoskeletal disorders we included AE showed no effective relief, as shown in Fig. 3B. Although we can see after looking back at each of the included studies, the results of most studies showed improvement of AE on patients' physical functioning due to LE. However, the combined analyses revealed that the results were not significant. This may be attributed to the limited amount of literature included or differences in the intensity of AE versus LE interventions.

### Quality of life

Regarding quality of life, twenty-two studies were included in the meta-analysis. Among them, eighteen studies [9, 11–13, 21–24, 27, 29, 32, 33, 35, 36, 39, 44–46] reported the comparison of AE and NE, six studies [11, 13, 26, 34, 43, 47] reported the comparison of AE and LE, and two studies [11, 13] compared three interventions.

**Fig. 4:**
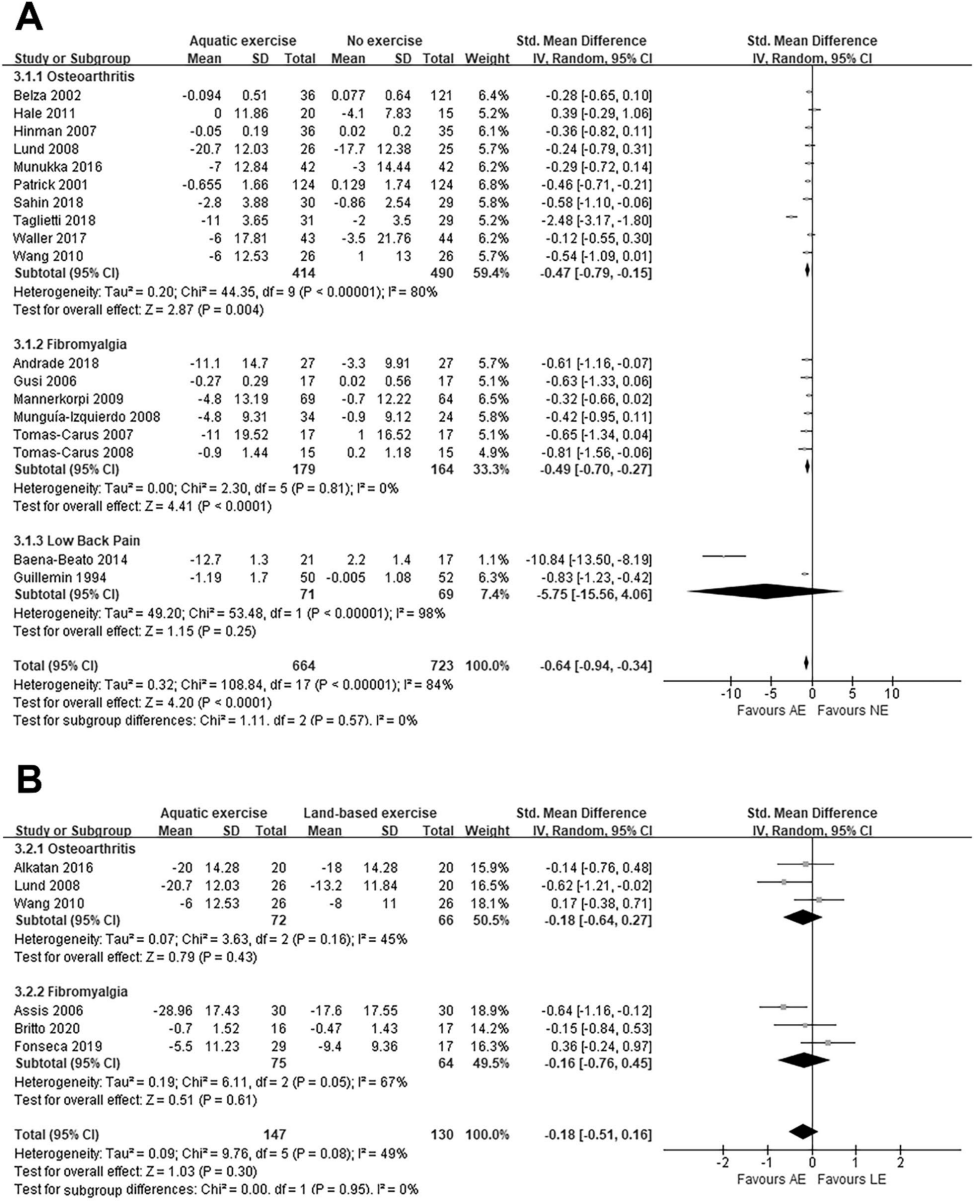
Forest plot of quality of life outcomes **A** AE versus NE; **B** AE versus LE

After the comparison of AE and NE, we could see that AE improved the quality of life of the participants (SMD: -0.64, *P* < 0.001), and after our subgroup analysis, it was clear that almost all experiments showed an effective improvement of quality of life by AE except for low back pain. The detailed results can be seen in Fig. 4A.

As with the previous outcomes, there was no significant difference in the improvement in quality of life between AE and LE (Fig. 4B).

### Publication bias and sensitivity analysis

To assess publication bias, the funnel plots of all outcome measures were obtained (Fig. S2-4). On visual inspection, all the funnel plots seem symmetrical with the effect estimates, and it indicated that the results are without significant publication bias. Additionally, sensitivity analyses were conducted to exclude each result, and the results proved the stability.

## Discussion

This is a systematic review and meta-analysis of whether AE has a positive impact on the treatment of chronic musculoskeletal disorders, the results of the study showed that patients who performed AE showed considerable improvements in pain, physical function, and quality of life compared to those who did NE. In the meantime, AE showed a more significant improvement in the vital indicator of pain compared to patients conducting LE but did not show a remarkable advantage in terms of physical function and quality of life. Nevertheless, our review of the included literature showed that overall, the initial number of participants was lower [26] and the rate of follow-up missed during the intervention was higher [11] in the LE group compared to AE, suggesting that patient engagement is higher in AE than in LE and that the effectiveness of an intervention is based on the efficacy of the intervention itself combined with patient engagement, so that subject engagement is critical in assessing the final effect. This is because even if an intervention is very effective, it will not show much of a benefit if the patient's willingness to participate is weak.

From the authors' investigation, there have been no previous studies on whether AE can improve chronic musculoskeletal disorders, thus this is the first systematic review and meta-analysis of this disease. Meanwhile, we selected important indicators such as pain, physical function, and quality of life, which have a high assessment value for patients' activities of daily living, to ensure the scientific accuracy of the study. Moreover, the total number of patients included in this study was 2200, and the advantage of a large population base makes the study results more credible. Besides, we included and summarized multiple types of chronic musculoskeletal disorders, which makes the study results more applicable to a wider population.

### Impact of AE on pain

The study used a pain assessment scale that allowed the assessors to determine whether AE had a positive impact on the improvement of pain values. The combined results showed that in a comparative analysis of AE versus NE and LE, AE was found to have a significantly better improvement in pain values than NE and LE. Also, the corresponding subgroup analysis showed that in all subgroups, the comparison between AE and NE showed a statistical difference, which can be interpreted in light of how AE affects the process of pain production. Pain is a sophisticated physiological phenomenon and as the main clinical manifestation in patients with osteoarthritis, fibromyalgia, etc., it is generated for complex reasons. In the normal population, the central nervous system balances the level of excitation and inhibition, so that no pain is generated, but in patients with chronic pain, this balance is disrupted [49]. In Baraniuk's study [50], it is known that the MEAP (Met-enk-Arg6-Phe7) in the cerebrospinal fluid of patients with fibromyalgia and low back pain compared to the normal group concentrations were significantly altered, and this suggests that altered levels of central nervous system opioid may cause or exacerbate fibromyalgia. Therefore, the consumption of opioids before and after the intervention can be used to determine the degree of pain reduction or worsening [51].

It is well established that physical exercise, a cost-effective and safe rehabilitation therapy, reduces pain levels by enhancing neurological 5-HT (5-hydroxytryptamine) neurotransmission, decreasing 5-HT transporter protein expression, and increasing 5-HT receptor expression through low-intensity aerobic training [52]. One step further, physical exercise in an aquatic environment may be more beneficial for pain relief. The pain-relieving effect of AE may stem from the combined effect of exercise, warm water, and buoyancy on thermoreceptors and mechanoreceptors [17], water pressure, water viscosity, and water temperature stimulate the senses during AE, promoting the triggering of thermoreceptors and mechanoreceptors and blocking the conduction of nociceptors (nociceptors are small-diameter nerve fiber endings that respond to the tissue environment) [53]. At the same time, the temperature and pressure of the water stimulate the skin, and while submerged in water, methionine encephalin plasma levels rise and reduce plasma levels of β-endorphin, corticotropin, and prolactin [53]. It deserves to be mentioned that the process of muscle activity produces several cytotoxic substances, the continuous accumulation of which activates sensitizes, or awakens nociceptors thereby producing pain, cytotoxic substances including histamine, serotonin, bradykinin, adrenaline, etc. [54], some research has reported increased levels of glutamate in fibromyalgia patients, which as a neurotransmitter transmitting pain stimulates the nociceptors, while the bradykinin stimulates the release of norepinephrine and prostaglandins to sensitize the nociceptors further. Hence, the improvement of pain can also be explained by the mechanism that the association of water pressure and temperature produces competing stimulation of nerve endings [55], thus being able to reduce injury from the periphery, and that hydrotherapy can also relax muscles, reduce their tension and decrease pain.

### Effect of AE on physical function

In assessing this indicator of patients' physical function, the majority of studies used the SF-36 (Medical Outcomes Study 36-Item Short-Form Health Survey) scale [12, 22, 28, 29, 31, 48], while some of them chose the WOMAC (Western Ontario and McMaster University Osteoarthritis Index) and FIQ (Fibromyalgia Impact Questionnaire) scales [30, 37, 44]. Because the assessment scales are much more similar, the results are also more accurate. Physical function is significant as an important indicator to evaluate the effect of AE on patient improvement. Patients with chronic musculoskeletal disorders show low physical function in several aspects, for instance, patients with fibromyalgia syndrome are prone to fatigue and dyspnea, which may be related to changes in the respiratory system, and according to clinical observations, patients have lower respiratory muscle endurance, inspiratory muscle strength, and chest mobility [56]. It was also found that the patient's heart rate was significantly elevated, cardiovascular sympathetic nerve activity showed increased, vagal nerve activity decreased, and the regulation of the sinus node was reduced, and thus, improving the patient's physical function was most significant in improving cardiovascular and respiratory function. Several studies have demonstrated that aerobic exercise can improve neurological disorders as well as cardiopulmonary function [57] and that the aquatic environment can reduce cardiovascular stress in patients, allowing for more intense training. A study by Zamunér et al*.* [58] demonstrated that aquatic therapy not only increased patients' aerobic capacity but also improved cardiac autoregulation; meanwhile, it was also reported in the paper that AE increased patients' oxygen uptake at rest and during exercise, as evidence of a certain degree of improvement in cardiopulmonary function [59].

For the subgroup analysis of physical function, AE compared with NE showed statistically significant differences in all subgroups. Therefore, this result further suggests that AE has a significant improvement in physical function compared to NE in most categories of chronic musculoskeletal disorders. While the comprehensive results of AE compared to LE showed no statistical difference, this result suggests that AE and LE are similar in terms of their effectiveness in improving the physical function of patients. When comparing the intervention methods of the studies in each group, it was found that more trials provided more differences in the training programs for AE and LE. The differences are not limited to the training period and intensity, and the variations in the training program will have an impact on the final results, so more research is needed to verify the final results of AE and LE.

### Impact of AE on quality of life

Quality of life is a very sophisticated metric that assesses a wide range of aspects, including patient mood, fatigue, perceived ability, and mental health, all of which can influence a patient's ability to live a normal life. Given the complexity of this index, the scales used to assess patients' quality of life varied across studies, and we, therefore, included the corresponding data by selecting scales with relatively similar assessment methods and criteria after due consideration. Quality of life is the best indicator of a patient's normal life, so the emphasis on improving the quality of life for patients is overwhelming. With AE intervention, warm water can put the patient's muscles in a relaxed state and reduce the pressure of gravity on the joints, in addition, exercise can promote the release of β-endorphin and dopamine in the patient's body [60], β-endorphin is a kind of endogenous morphine-like substance in the human body, which has a strong analgesic effect, dopamine is the most abundant catecholamine neurotransmitter in the brain, which transmits signals of excitement as well as happiness, and it plays an important role in human movement and learning. The increased release of these two substances has a calming and analgesic effect on the subjects, as well as relieving anxiety and achieving the effects of antidepressants. In the meantime, the aquatic environment provides a relaxing and comfortable atmosphere for patients, which can increase their pleasure [28], relieving their depression or irritability caused by their disease and the pain it brings. Besides, water exercise can also be considered as a kind of water immersion method, which affects some physiological responses of the body, such as changing the fluid in the cells and blood vessels, reducing edema, increasing blood flow by diastole, and increasing cardiac output, which can relieve fatigue and have psychological benefits for the participants [61].

The meta-analysis of the quality of life indicator showed a significant improvement in AE compared to NE, but no statistically significant difference when compared to LE, which may be due to the short intervention period in some of the studies resulting in similar changes in various aspects of the subjects, as a result, no significant difference could be revealed between the two programs. Meanwhile, we also found statistics showing that patients with osteoarthritis have lower physical mobility compared to the general population, and nearly 50% of patients will be reluctant to perform additional exercise training due to pain [14, 62]. So there is a problem of low patient willingness to treat and low participation rate, while the level of participation of patients in both groups will also affect the final results. Subgroup analysis showed that the low back pain subgroup showed no statistical difference in the AE versus NE comparison, and this is most likely due to the small number of literature with two articles, which makes it biased from the actual situation.

### Limitations and future research

There are several limitations of this systematic review and meta-analysis. Firstly, this study only included RCTs published in English, and some high-quality articles published in languages other than English were excluded, so future meta-analyses should include these excluded high-quality articles as well as some valuable studies that may not have been published yet; secondly, because the number of included literature within some subgroups was found to be small when subgroup analysis was conducted in this study, which could lead to a large heterogeneity in some subgroup analysis and make the results of meta-analysis differ from the actual results. Thus, the number of included literature should be increased as much as possible in future studies to further improve the reliability of the study; the third is that the intervention protocols are not identical across studies, so the intervention intensity, as well as the intervention period, can vary, both of which can affect the final intervention effect; in the fourth, there is a placebo effect for aquatic therapy, and most of the literature included in this paper lacked a placebo control, so the placebo effect could not be excluded [33].

Considering the limitations mentioned above, future meta-analyses should carefully consider the period of interventions included in the paper, either too long or too short could adversely affect the final results, and the intensity of interventions needs to be kept as similar as possible between studies, in addition to further evaluation of the placebo effect of AE to determine whether it affects the results and to what extent. In other words, most of the AE therapies in the studies included in this article require the supervision of professional physiotherapists and the development of exercise programs for patients that are appropriate to their physical conditions, but there is still much space to improve the popularity of AE because of the limited space and related exercise facilities available for AE. Therefore, in the foreseeable future, more achievable and affordable AE programs should be developed for the stakeholders to increase their popularity, allowing patients to receive treatment in a safer and more comfortable environment, reducing pain, and improving their quality of life. Moreover, since AE applies to a wide range of groups and there are some differences in treatment measures and contraindications among different diseases, it is recommended that future studies should address individual differences and disease differences. For instance, AE showed significant improvement in physical function compared to NE in patients with osteoarthritis and fibromyalgia, but not in patients with low back pain. Therefore, it is essential to develop exercise programs for different patients that match their intervention objectives, physical conditions, and personal activity habits to maximize patient participation and intervention effects [63].

Given the positive relationship established in this meta-analysis that AE has a positive effect on the treatment of chronic musculoskeletal disorders compared to NE, future research is required to explore whether there is a positive effect of AE in areas other than the indicators studied here. Moreover, AE has been found to have a significant improvement in pain compared to LE in patients with chronic musculoskeletal disorders, but the efficacy has not yet been demonstrated in terms of physical function and quality of life, so more studies are needed to compare its efficacy in other aspects.

## Conclusions

The results of the study showed that AE significantly improved pain, physical function, and quality of life in patients with chronic musculoskeletal disorders compared to NE; when compared to LE, AE was only found to improve pain in patients. Considering the clinical application of this rehabilitation tool, more long-term clinical trials are still needed to further confirm the positive effects and improvement mechanisms of AE and the more existential advantages compared to LE as a treatment measure.

### Supplementary Information

**Additional file 1: Table S1** The detailed search strategy and results.** Figure S1** The quality assessment of RCTs. **A** Risk-of-bias item presented as percentages across RCTs for population with diseases; **B** Judgments about risk-of-bias item for each RCTs. + indicates low risk, ? indicates unclear risk, − indicates high risk.** Figure S2** Funnel plot of pain outcomes. **A** AE versus NE; **B**AE versus LE. **Figure S3** Funnel plot of physical function outcomes. **A** AE versus NE; **B** AE versus LE. **Figure S4** Funnel plot of quality of life outcomes **A** AE versus NE; **B**AE versus LE.

## Data Availability

The datasets used and/or analyzed during the present study are available from the corresponding author upon reasonable request.

## Abbreviations

AE: Aquatic exercise
RCT: Randomized controlled trial
LE: Land-based exercise
NE: No exercise
SD: Standard deviation
CI: Confidence interval
SE: Standard error
SEM: Standard error of the mean
SMD: Standardized mean difference
MEAP: Met-enk-Arg6-Phe7
5-HT: 5-Hydroxytryptamine
SF-36: Medical Outcomes Study 36-Item Short-Form Health Survey
WOMAC: Western Ontario and Mcmaster University Osteoarthritis Index
FIQ: Fibromyalgia Impact Questionnaire
