# Supplementary material for: Efficacy of aquatic exercise in chronic musculoskeletal disorders: a systematic review and meta-analysis of randomized controlled trials
Source: J Orthop Surg Res. 2023 Dec 8;18:942. doi: 10.1186/s13018-023-04417-w (PMC10704680; doi:10.1186/s13018-023-04417-w)
Supplement: Supplementary file 1 — Additional file 1: Table S1 The detailed search strategy and results. Figure S1 The quality assessment of RCTs. A Risk-of-bias item presented as percentages across RCTs for population with diseases; B Judgments about risk-of-bias item for each RCTs. + indicates low risk, ? indicates unclear risk, − indicates high risk. Figure S2 Funnel plot of pain outcomes. A AE versus NE; BAE versus LE. Figure S3 Funnel plot of physical function outcomes. A AE versus NE; B AE versus LE. Figure S4 Funnel plot of quality of life outcomes A AE versus NE; BAE versus LE. [file 13018_2023_4417_MOESM1_ESM.pdf]

Table of Contents

SUPPLEMENTAL TABLE **2**
Table S1 .... **2-3**
SUPPLEMENTAL FIGURES **4**
Figure S1 .... **4**
Figure S2 .... **5**
Figure S3 .... **6**
Figure S4 .... **7**

SUPPLEMENTAL TABLE

Table S1. The detailed search strategy and results

Pubmed:

Set #	Terms	Results
1	(hydrotherapy OR whirlpool baths OR (bath, whirlpool) OR (baths, whirlpool) OR whirlpool bath OR watsu therapy OR (therapy, watsu) OR water shiatsu OR (shiatsu, water) OR watershiatsu)	21547
2	(aquatic therapy OR (therapy, aquatic) OR aquatic exercise therapy OR (exercise therapy, aquatic) OR (therapy, aquatic exercise) OR water exercise therapy OR (exercise therapy, water) OR (therapy, water exercise) OR pool therapy OR (therapy, pool) OR ai chi therapy OR (therapies, ai chi) OR (therapy, ai chi) OR water tai chi therapy)	17101
3	1 OR 2	38262
4	(musculoskeletal OR musculoskeletal pain OR musculoskeletal diseases OR musculoskeletal disorders OR chronic musculoskeletal diseases OR chronic musculoskeletal disorders)	1272726
5	3 AND 4	2853
6	not (systematic review OR meta-analysis OR case reports OR editorial OR letter OR comment)	2119

Cochrane library:

Set #	Terms	Results
1	hydrotherapy:ti,ab OR 'aquatic therapy':ti,ab OR 'aquatic exercise':ti,ab	1378
2	'chronic musculoskeletal diseases':ti,ab OR 'musculoskeletal diseases':ti,ab	9,293
3	1 AND 2	56
4	NOT ('case report'/exp OR 'case study'/exp OR 'editorial'/exp OR [editorial]/lim OR 'letter'/exp OR [letter]/lim OR 'note'/exp OR [note]/lim OR [conference abstract]/lim OR 'conference abstract'/exp OR 'conference abstract'/it OR 'systematic review':ti OR 'meta-analysis':ti OR 'systematic review'/exp OR 'meta analysis'/exp) AND [humans]/lim	54

Web of Science (via Clarivate):

Set #	Terms	Results
1	TS=(hydrotherapy OR “aquatic therapy” OR “pool therapy” OR “water exercise therapy”)	134,699
2	TS=("chronic musculoskeletal diseases " OR "musculoskeletal diseases")	6,005
3	1 and 2	491
4	Refined by: [excluding] DOCUMENT TYPES: (EDITORIAL MATERIAL OR REVIEW OR MEETING ABSTRACT OR LETTER OR PROCEEDINGS PAPER)	264

Embase (via Elsevier):

Set #	Terms	Results
1	hydrotherapy:ti,ab OR 'aquatic therapy':ti,ab OR 'aquatic exercise':ti,ab	620,181
2	'chronic musculoskeletal diseases':ti,ab OR 'musculoskeletal diseases':ti,ab	36,814
3	1 AND 2	12,834
4	NOT ('case report'/exp OR 'case study'/exp OR 'editorial'/exp OR [editorial]/lim OR 'letter'/exp OR [letter]/lim OR 'note'/exp OR [note]/lim OR [conference abstract]/lim OR 'conference abstract'/exp	354

	OR 'conference abstract'/it OR 'systematic review':ti OR 'meta-analysis':ti OR 'systematic review'/exp OR 'meta analysis'/exp) AND [humans]/lim	

SUPPLEMENTAL FIGURES

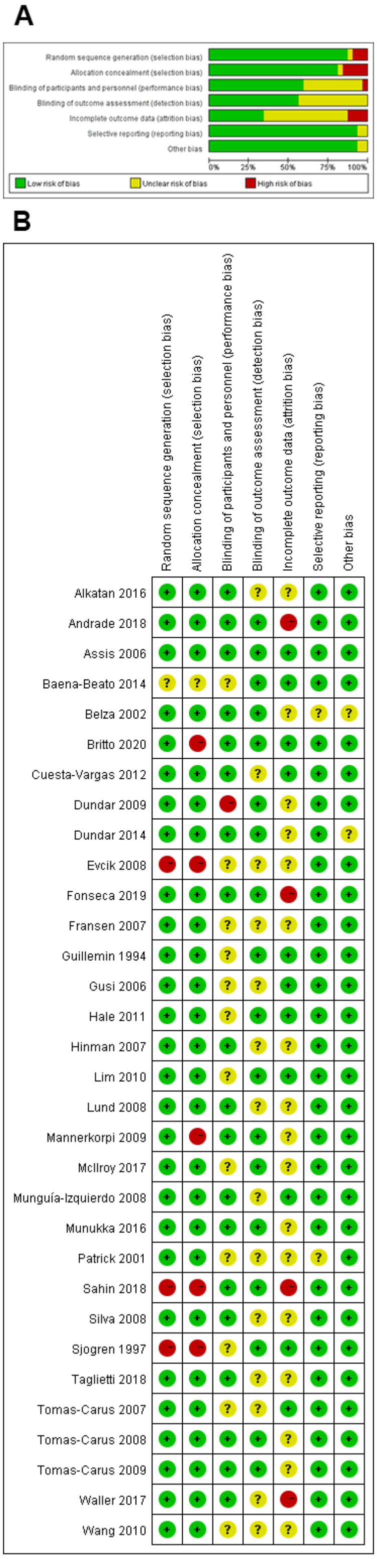

Figure S1. The quality assessment of RCTs

(A)Risk-of-bias item presented as percentages across RCTs for population with diseases; (B)Judgments about risk-of-bias item for each RCTs. + indicates low risk,? indicates unclear risk, − indicates high risk.

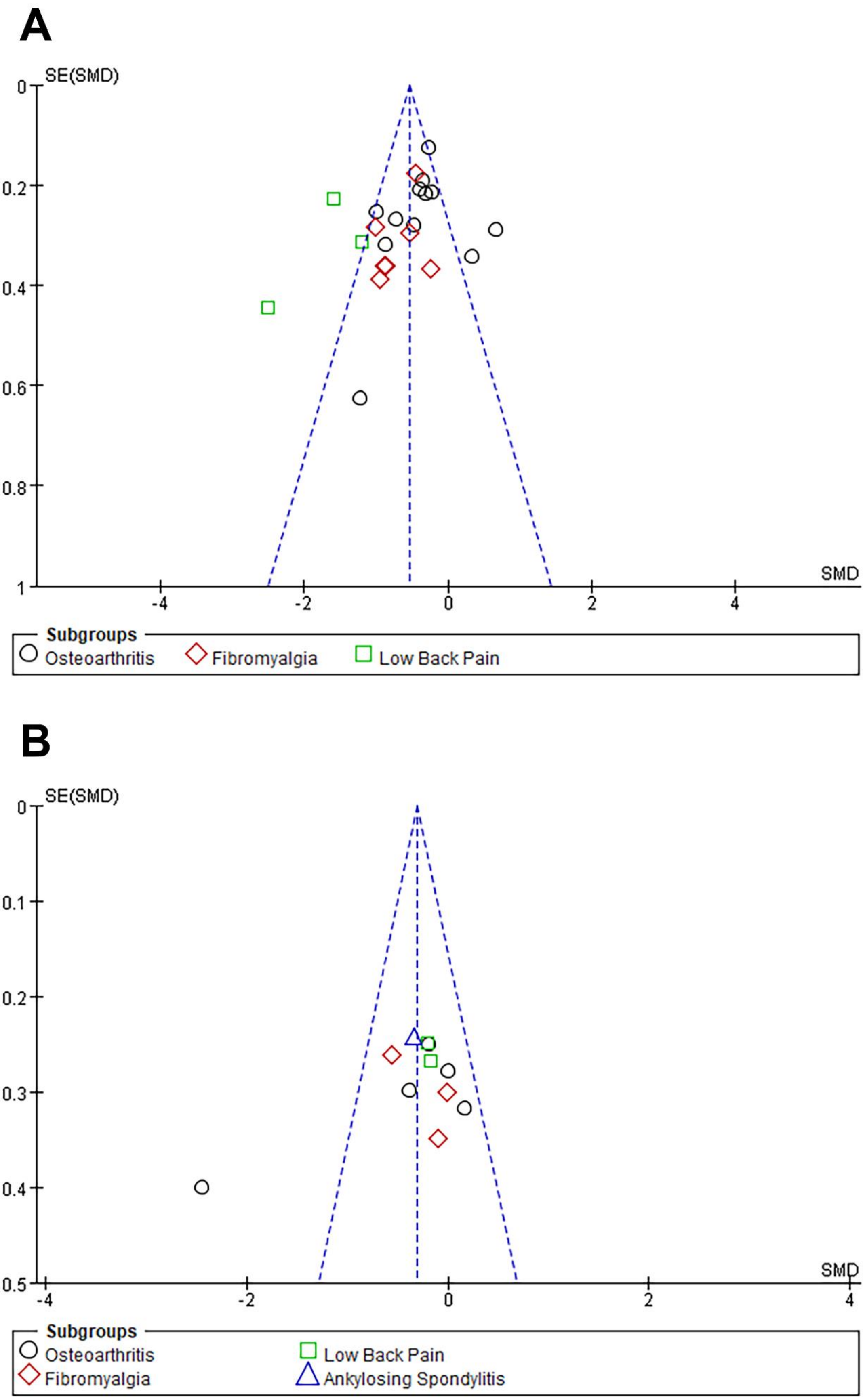

Figure S2. Funnel plot of pain outcomes

(A) AE versus NE; (B) AE versus LE.

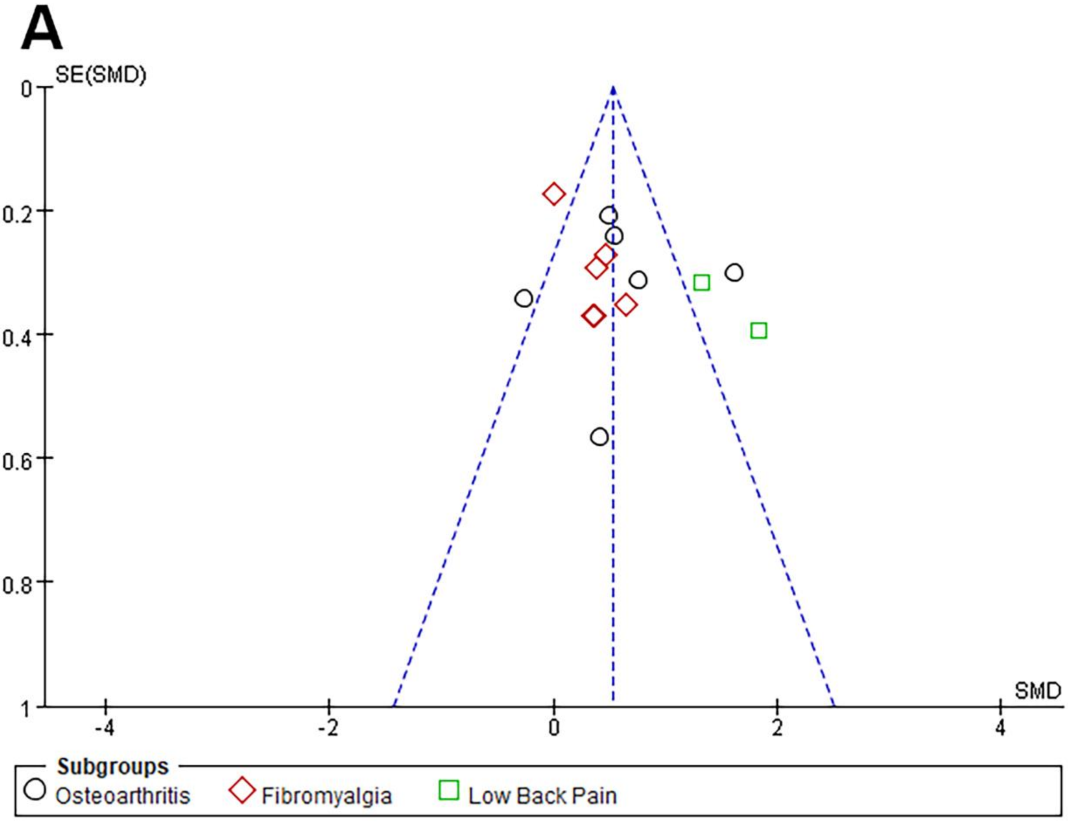

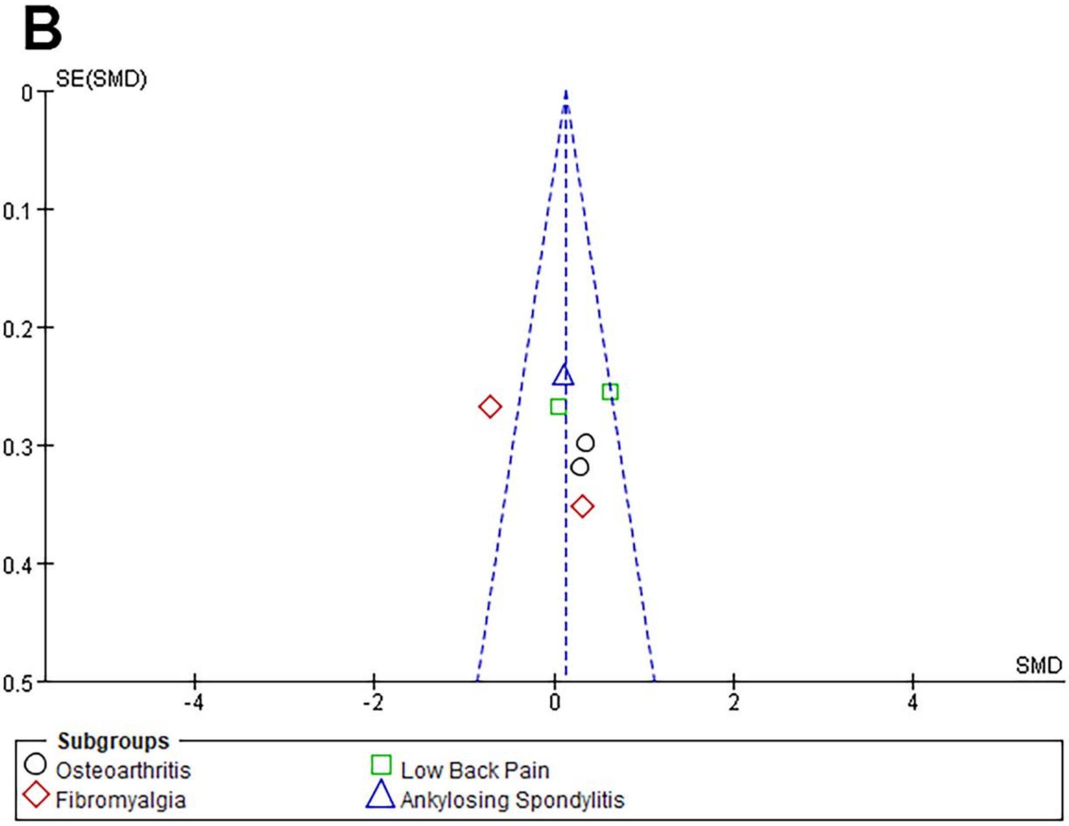

Figure S3. Funnel plot of physical function outcomes

(A) AE versus NE; (B) AE versus LE.

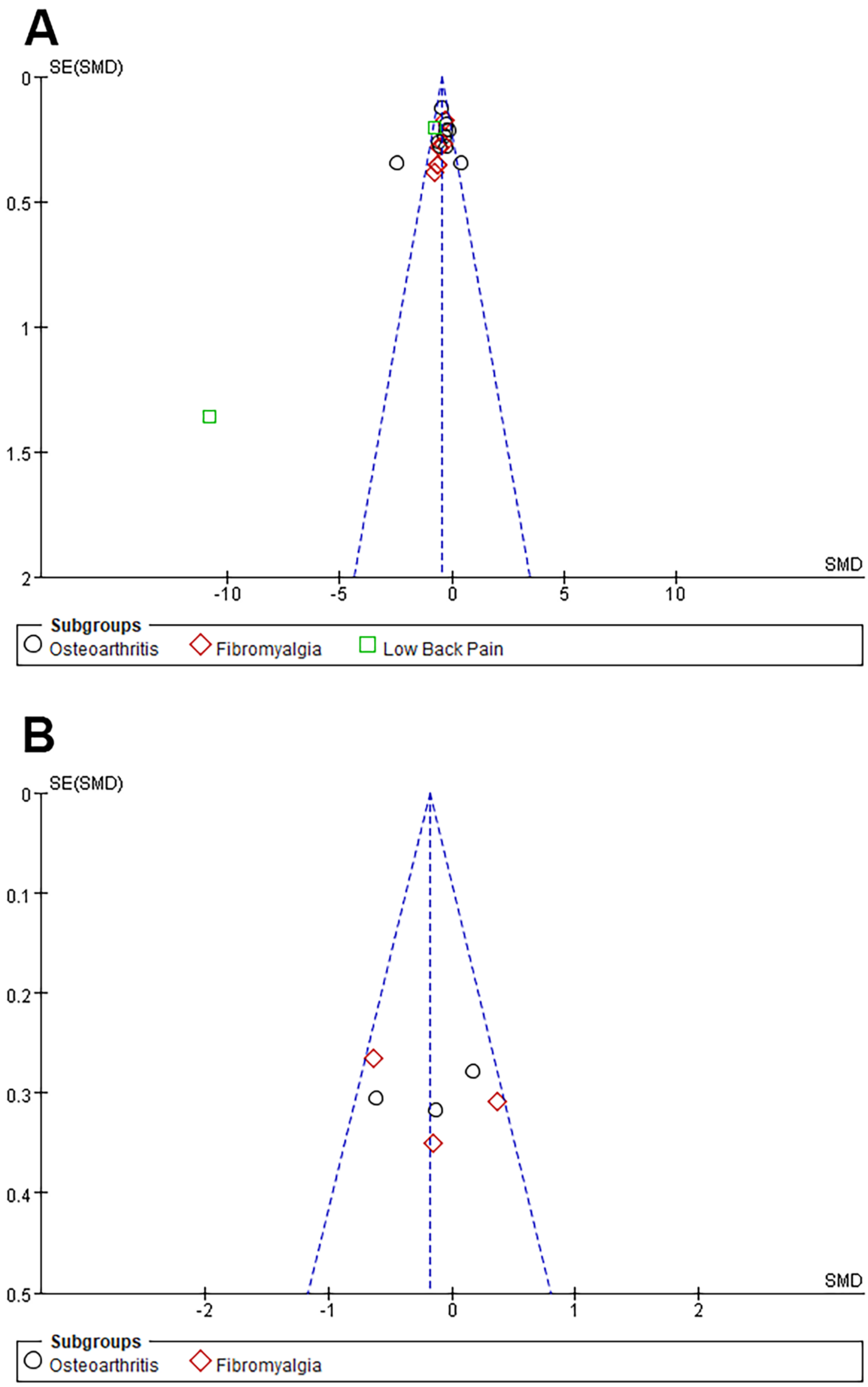

Figure S4. Funnel plot of quality of life outcomes

(A) AE versus NE; (B) AE versus LE.
